# SP1 induced long non-coding RNA AGAP2-AS1 promotes cholangiocarcinoma proliferation via silencing of CDKN1A

**DOI:** 10.1186/s10020-020-00222-x

**Published:** 2021-02-01

**Authors:** Hao Ji, Juan Wang, Binbin Lu, Juan Li, Jing Zhou, Li Wang, Shufen Xu, Peng Peng, Xuezhen Hu, Keming Wang

**Affiliations:** 1 Department of Oncology, Second Affiliated Hospital, Nanjing Medical University, Nanjing, 210000 Jiangsu People’s Republic of China; 2 The Second Clinical Medical College of Nanjing Medical University, Nanjing, China; 3 Jiangsu Provincial Hospital of Traditional Chinese Medicine, Nanjing, China; 4 Department of Radiology, Affiliated Hospital of Nanjing University of Chinese Medicine, Nanjing, 210000 Jiangsu People’s Republic of China

**Keywords:** LncRNA, AGAP2-AS1, CCA, EZH2, Biomarker apoptosis

## Abstract

**Background:**

LncRNA can regulate gene at various levels such as apparent genetics, alternative splicing, and regulation of mRNA degradation. However, the molecular mechanism of LncRNA in cholangiocarcinoma is still unclear. This deserves further exploration.

**Methods:**

We investigated the expression of AGAP2-AS1 in 32 CCA tissues and two CCA cell lines. We found a LncRNA AGAP2-AS1 which induced by SP1 has not been reported in CCA, and Knockdown and overexpression were used to investigate the biological role of AGAP2-AS1 in vitro. CHIP and RIP were performed to verify the putative targets of AGAP2-AS1.

**Results:**

AGAP2-AS1 was significantly upregulated in CCA tumor tissues. SP1 induced AGAP2-AS1 plays an important role in tumorigenesis. AGAP2-AS1 knockdown significantly inhibited proliferation and caused apoptosis in CCA cells. In addition, we demonstrated that AGAP2-AS1 promotes the proliferation of CCA.

**Conclusions:**

We conclude that the long non-coding RNA AGAP2-AS1 plays a role in promoting the proliferation of cholangiocarcinoma.

## Background

Cholangiocarcinoma (CCA) is the uncontrollable transformation of biliary duct cells derived from intrahepatic and extrahepatic bile ducts (Blechacz 2017; Pickersgill et al. 2019). In recent years, the frequency of cholangiocarcinoma has increased gradually (Siegel et al. 2018). The cause of CCA may be associated with bile duct stones, primary sclerosing cholangitis and other diseases. Surgery, radiotherapy, chemotherapy, and other methods can be used for treatment, but the prognosis is poor. Because there are no specific biomarkers or special clinical manifestations, CCA is usually diagnosed as advanced (Acquisto et al. 2018; Fung and Tabibian 2019). Molecular targeting drugs have no effect on CCA, mainly because the molecular mechanism of CCA is not clear (Blechacz 2017; Komuta and Yeh 2019). In order to improve the diagnosis rate and cure rate of CCA, we need to further explore and clarify the molecular pathogenesis of CCA.

Long non-coding RNA (lncRNA) are transcripts with a length of more than 200 nucleotides and no protein coding function (Chen et al. 2018a). With the development of the second generation sequencing technology, more and more lncRNA functions have been discovered and become the focus of research (Bayes et al. 2012; Liu et al. 2018). Studies on the expression, recruitment of chromatin trimmings, regulation of X chromosome inactivation (XIST) (Zhu et al. 2018), genomic imprinting (H19) (Luo et al. 2018), protein folding and protein activity, other processes have revealed that lncRNAs can function carcinogenic or tumor suppressor genes in tumor development (Zhang and Ho 2019). Many studies have shown abnormal lncRNA expression in tumors (Chen et al. 2018a; Zhao et al. 2018b, c; Cui et al. 2018). The methods used for analyzing lncRNA include expression chip technology, RNA sequencing technology, and so on, using tissue, blood, urine, and saliva samples (Perron et al. 2017). LncRNA is expected to be a new marker for tumor diagnosis and prognosis. However, the mechanism of lncRNAs in cholangiocarcinoma is unclear.

To investigate the dysregulated lncRNAs in cholangiocarcinoma, we screened the 36 cancer tissue and 9 normal tissue in TCGA database and found that lncRNA AGAP2-AS1 is high expression in tumor tissues,likely involved in cholangiocarcinoma progression. LncRNA AGAP2-AS1 is localized on human chromosome 12, and has not been previously reported in cholangiocarcinoma. Then we verified the expression of AGAP2-AS1 in 32 pairs of clinical samples and two CCA cell lines by qRT-PCR. It was proved that it was highly expressed in both cancer tissues and cancer cells.In addition, we also found that transcription factor SP1 can induce the high expression of AGAP2-AS1. In vitro and vivo experiments indicate that knockdown of AGAP2-AS1 inhibits CCA cell proliferation, colony formation, and promotes apoptosis. In addition, we also studied the molecular mechanism of AGAP2-AS1 in CCA cells and found potential targets. Subsequent experiments explored the association of AGAP2-AS1 and CDKN1A, revealing that AGAP2-AS1 can negatively regulate CDKN1A expression. Overall, SP1 induced-AGAP2-AS1 predicts poor prognosis in patients with CCA and promotes CCA cell proliferation, in part by inhibiting CDKN1A expression.

## Methods

### Tissue collection

In this study, 32 intrahepatic tumor tissues were collected from CCA patients undergoing surgery in the Second Affiliated Hospital of Nanjing Medical University. All patients had not received treatment before. All operations were approved by the Research Ethics Committee of Nanjing Medical University of China and met the requirements.

### Cell lines and culture conditions

Human CCA cell lines (RBE and HUCCT1) and normal biliary epithelial cells (HIBEpic) were obtained from the American Type Culture Collection (Manassas, VA) and cultured in In DMEM medium containing 10% fetal bovine serum (DMEM; Invitrogen). The cells were cultured in an incubator at 37° C, 5% CO^2^, and 90% relative humidity, and the cells were adherently grown. Fresh medium was replaced every 2–3 day(s) and passaged when the cell fusion reached 80–90%.

### RNA extraction and qPCR assays

We extracted total RNA from tissues or cultured cells by TRIzol reagent (Invitrogen, Carlsbad, CA). Reverse transcription of total RNA into cDNA by reverse transcription reagents (Takara, Dalian, China). Real-time PCR was analyzed by SYBR Premix ExTaq (Takara, Dalian, China). The results were normalized to GAPDH expression. Performing real-time PCR assays on the ABI 7500 system and collect data from the instrument. The primer sequences are listed in Additional file 1: Table S1.

### Transfection of CCA cells

The siRNA of AGAP2-AS1 (AGAP2-AS1 1# and 2#), EZH2, CDKN1A, SP1,and scrambled negative control (NC) were purchased from Invitrogen and transfected into cells using Lipofectamine 2000 (Invitrogen, USA). shRNA is used for projects that require long-term experiments, such as clone formation and subcutaneous injection of animals. The interference sequences used are listed in Additional file 1: Table S1. Plasmid vectors were from general (Shanghai, China) and extracted using a DNA Midiprep kit (Qiagen, Hilden, Germany) and transfected into cells by Fugene reagent (Roche, Basel, Switzerland).

### Cell proliferation assays

Cell proliferation assays were performed using Cell Counting Kit-8 (CCK8) (Promega). RBE and HUCCT1 cells transfected with si-AGAP2-AS1 for 24 h(s), were seeded in 96-well plates and incubated at 37 °C under 5% CO_2_. Relative cell growth was measured every 24 h(s). After incubation with CCK8 solution for 2 h(s), the absorbance was measured at 450 nm. Colony formation assays were performed to monitor CCA cell clonality. RBE and HUCCT1 cells transfected with sh-AGAP2-AS1 were placed in a six-well plate and replaced with medium containing 10% FBS for approximately 14 day(s) with the medium replaced every 5 day(s) for colony formation assays. After 14 day(s), the medium was discarded and washed with PBS, fixed with methanol, stained with 0.1% crystal violet (Sigma-Aldrich).

### Flow cytometric analysis

RBE and HUCCT1 cells transfected with si-AGAP2-AS1 were harvested after 48 h. After staining with FITC-Annexin V and propidium iodide (PI) using the FITC Annexin V Apoptosis Detection Kit (BD Biosciences), cells were analyzed by flow cytometry (FACScan; BD Biosciences) by CellQuest software (BD Biosciences). The cells were divided into living cells, dead cells, early apoptotic cells and apoptotic cells, and the relative proportion of early apoptotic cells in each experiment was compared with that of the control group.

### Animal experiments

Male athymic BALB/c nude mice (4 weeks old) were maintained under pathogen free conditions. 1.5 × 10^6^ cells were suspended in 0.1 ml serum-free medium, mixed with 0.1 ml ECMgel, injected subcutaneously into the back of nude mice, and re-injected with the same number of cells at the same site three days later. RBE cells stably transfected with sh-AGAP2-AS1 or an empty vector were injected subcutaneously into the axilla of the mouse, and the tumor volume was measured every 3 day(s). Eighteen days after the injection, the mice were sacrificed and the volume and mass of each subcutaneously growing tumor were examined. The formula for calculating tumor volume is: (L × W2) / 2, where L is the maximum length of the tumor and W is the maximum width of the tumor. Tumor tissues were used for qPCR analysis of AGAP2-AS1 levels, H&E staining and immunostaining of Ki-67 protein. The program was approved by the Animal Experimental Ethics Committee of Nanjing Medical University.

### Subcellular fractionation location

Separation of nuclear and cytoplasmic fractions was performed using the PARIS kit (Life Technologies) according to the manufacturer's protocol.

### Fluorescence in situ hybridization

RBE cells were seeded in 24-well plates, and when the cells accounted for 40% of the area, the medium was discarded. The cells were washed with PBS, fixed in 4% formaldehyde for 10 min(s), and washed again with PBS. Treatment with PBS containing 0.5% Triton X-100 was followed by three washes with PBS. The pre-hybridization solution was then added, and the probe-containing hybridization solution was added overnight. After washing with 4×, 2×, and 1× SSC, DAPI staining was performed. The RNA FISH probe was designed and synthesized by Ribobio (Guangzhou, China).

### RNA immunoprecipitation (RIP) assay

We performed RIP experiments using the Magna RIP RNA Binding Protein Immunoprecipitation Kit (Millipore, Billerica, MA, USA). HUCCT1 and RBE cells were lysed in complete RIP lysis buffer and whole cell extracts were incubated with beads. The beads were then washed with a wash buffer containing 0.1% SDS/0.5 mg/ml protease. A qRT-PCR assay was performed corresponding to the purified RNA to detect the presence of AGAP2-AS1. Antibodies for RIP determination of EZH2, Goods number: 17-662. Control IgG antibodies from Millipore (Billerica, MA, USA).

### Chromatin immunoprecipitation (CHIP) assays

HUCCT1 and RBE cells were treated with formaldehyde and incubated for 10 min to generate DNA–protein crosslinks. The cell lysate was then sonicated to generate a 200–300 bp chromatin fragment and immunoprecipitated with EZH2 or H3K27me3 specific antibody, Goods number: 17-662(Millipore, Billerica, MA, USA) or IgG as a control. After the precipitated chromatin was recovered, qRT-PCR analysis was performed.

### Luciferase reporter assays

We used JASPAR (https://jaspar.gener0eg.net/) online database to predict potential transcription factor of AGAP2-AS1 promoter regions, and several SP1 binding motifs were identified. The AGAP2-AS1 promoter region (2000 bp) was inserted into a pGL3-basic vector (Promega, Madison, WI, USA). The Dual-Luciferase Assay Kit following manufacturer’s protocol.

### Western blot analysis and antibodies

We isolated cell protein lysates using 10% sodium dodecyl sulfate polyacrylamide gel electrophoresis (SDS-PAGE). They were transferred to a 0.22 mm NC membrane (Sigma) and then incubated with specific antibodies. The ECL chromogenic substrate was used for quantification by densitometry (Quantity One software; Bio-Rad). Anti-CDKN1A was purchased from Abcam, AB109520, Dilution concentration 1 × 5000 (Hong Kong, China).

### Immunohistochemical (IHC) analysis

Xenograft tumor tissue samples were stained with H&E and immunostained for Ki67. Anti-Ki67 was obtained from Santa Cruz Biotechnology (Dallas, TX, USA). IHC staining results were professionally analyzed.

### Statistical analysis

We used SPSS software for statistical analysis (SPSS, Inc., Chicago, IL, USA). Clinical pathology data were analyzed by chi-square exact testing. All data were expressed as the means ± SD (standard deviation), and analyzed using the Student’s t test to compare two groups of in vitro and in vivo data Results are reported as mean ± standard deviation. Statistical significance was specified as P < 0.05 (*) or P < 0.01 (**).

## Results

### AGAP2-AS1 is upregulated in human CCA tissues

**Fig. 1:**
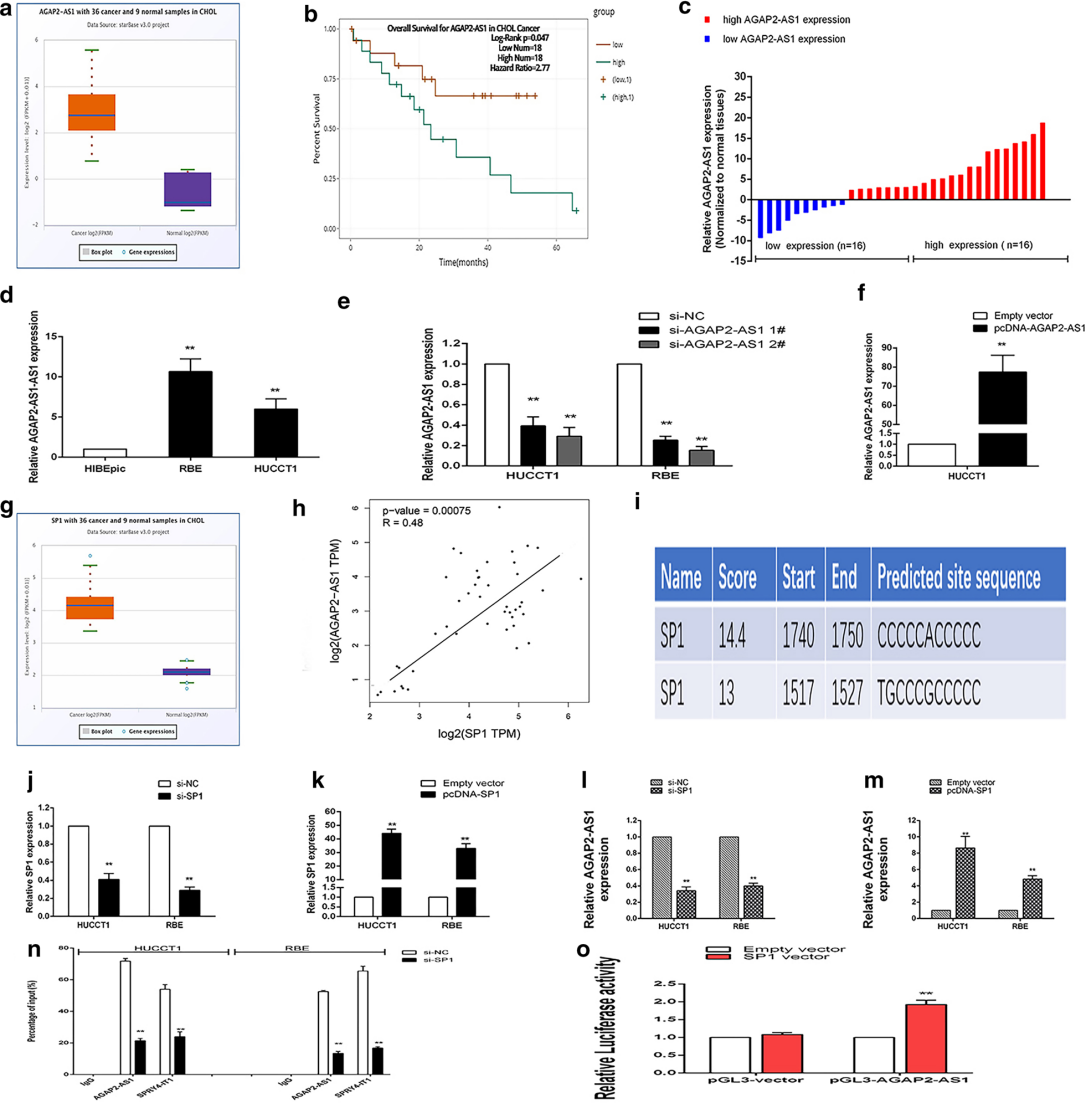
SP1 induces high AGAP2-AS1 expression in CCA. **a**, **b** Relative expression of AGAP2-AS1 in CCA tissues compared with the corresponding adjacent normal tissues (n = 32), and classification into two groups. **c** Kaplan–Meier overall survival curves based on AGAP2-AS1 expression level. **d** Analysis of AGAP2-AS1 expression levels in CCA cell lines (HUCCT1 and RBE) by qPCR. **e**, **f** qRT-PCR analysis of AGAP2-AS1 expression of HUCCT1 and RBE cells with si-AGAP2-AS1 1, 2, pcDNA-AGAP2-AS1, or the negative control. **g** JASPR prediction of SP1 binding sites in the AGAP2-AS1 promoter regions. **h** SP1 knockdown and overexpression efficiency. **i** Effect of knockdown or overexpression of SP1 on AGAP2-AS1. **j** CHIP for SP1 in the promoter region of AGAP2-AS1. **k** Luciferase reporter gene verification. Representative images and data based on three independent experiments. Bars: ± sd, *P < 0.05, **P < 0.01

We first analyzed the expression level of AGAP2-AS1 of 36 cancer tissue and 9 normal tissue in TCGA database ( https://gepia.cancer-pku.cn/). Compared with normal tissues, the expression of AGAP2-AS1 was higher in CCA tissues (Fig. 1a). According to the survival analysis of the database, it was also shown that patients with high expression of AGAP2-AS1 had a shorter survival time (Fig. 1b) (https://starbase.sysu.edu.cn/).AGAP2-AS1) expression levels were determined in 32 pairs of paired CCA tumor tissues and adjacent tissues using qRT-PCR and normalized to GAPDH. In addition, in order to assess the effect of AGAP2-AS1 on the difference in CCA prognosis, 32 CCA patients were divided into two groups, relative to the median ratio of AGAP2-AS1 expression in tumor tissues: relatively high AGAP2-AS1 group and relative Lower AGAP2-AS1 group (Fig. 1c). Table 1 summarizes the main details of patients with CCA. Our results suggest that AGAP2-AS1 is an unfavorable prognostic factor in patients with CCA.

**Table 1:** Relationships between AGAP2-AS1 expression and clinicopathological characteristics of CCA patients

Characteristics	AGAP2-AS1		P
	Low expression (n=16)	High expression (n=16)	P chi-squared test P-value
Age (years)			
≤ 60	8	7	0.724
> 60	8	9	
Gender			
Male	6	7	0.72
Female	10	9	
Tumor size (cm)			
≤ 5	11	4	0.013*
> 5	5	12	
Histological			
Middle or high	14	3	0.0001**
Low or undiffer	2	13	
TNM stage			
I, II	10	3	0.0012**
III, IV	6	13	
Lymph node metastasis			
Positive	2	12	0.0004**
Negative	14	4	

* P < 0.05

** P < 0.01

### AGAP2-AS1 expression regulation

To investigate the biological function of AGAP2-AS1 in CCA cells, we chosed RBE and HUCCT1 cell lines for further study because the expression of AGAP2-AS1 were higher than normal biliary epithelial cell lines (HIBEpic) (Fig. 1d). Next, we designed two different AGAP2-AS1 short interfering RNAs (siRNAs) and transfected them into two CCA cell lines (Fig. 1e). The overexpression plasmid was transfected into HUCCT1 cells with relatively low expression levels of AGAP2-AS1, and transfection efficiency was determined by qRT-PCR (Fig. 1f). Therefore, si-AGAP2-AS1 1, 2 and pcDNA-AGAP2-AS1 were used for all subsequent AGAP2-AS1 knockdown or overexpression experiments.

### SP1 induces high expression of AGAP2-AS1

According to the article and prediction, we found that the transcription factor SP1 may induce the high expression of AGAP2-AS1 (Dong et al. 2018). According to the starbase database based on TCGA database analysis (https://starbase.sysu.edu.cn/), we found that SP1 was highly expressed in cholangiocarcinoma tissues (Fig. 1g). And it was positively correlated with the expression of AGAP2-AS1 (Fig. 1h). In order to verify that SP1 induces AGAP2-AS1, we are predicting the SP1 binding site On the JASPR website (https://jaspar.binf.ku.dk) (Fig. 1i). we first designed the SP1 interference sequence and overexpression plasmid, and verified by qRT-PCR (Fig. 1 j–k). SP1 is a common upstream transcription factor (Chen et al. 2018b; Xu et al. 2018). We knocked down/overexpressed SP1 in CCA cells and found that AGAP2-AS1 changed accordingly (Fig. 1l, m). Next, we verify this conclusion by designing primers for the promoter region of the upstream sequence 2000 bp of AGAP2-AS1 and conducting CHIP experiments, we inserted the AGAP2-AS1 promoter region into the PGL3 vector, dual luciferase experiments also proved this conclusion (Fig. 1n, o).

### AGAP2-AS1 knockdown inhibits CCA cell proliferation, induces apoptosis

**Fig. 2:**
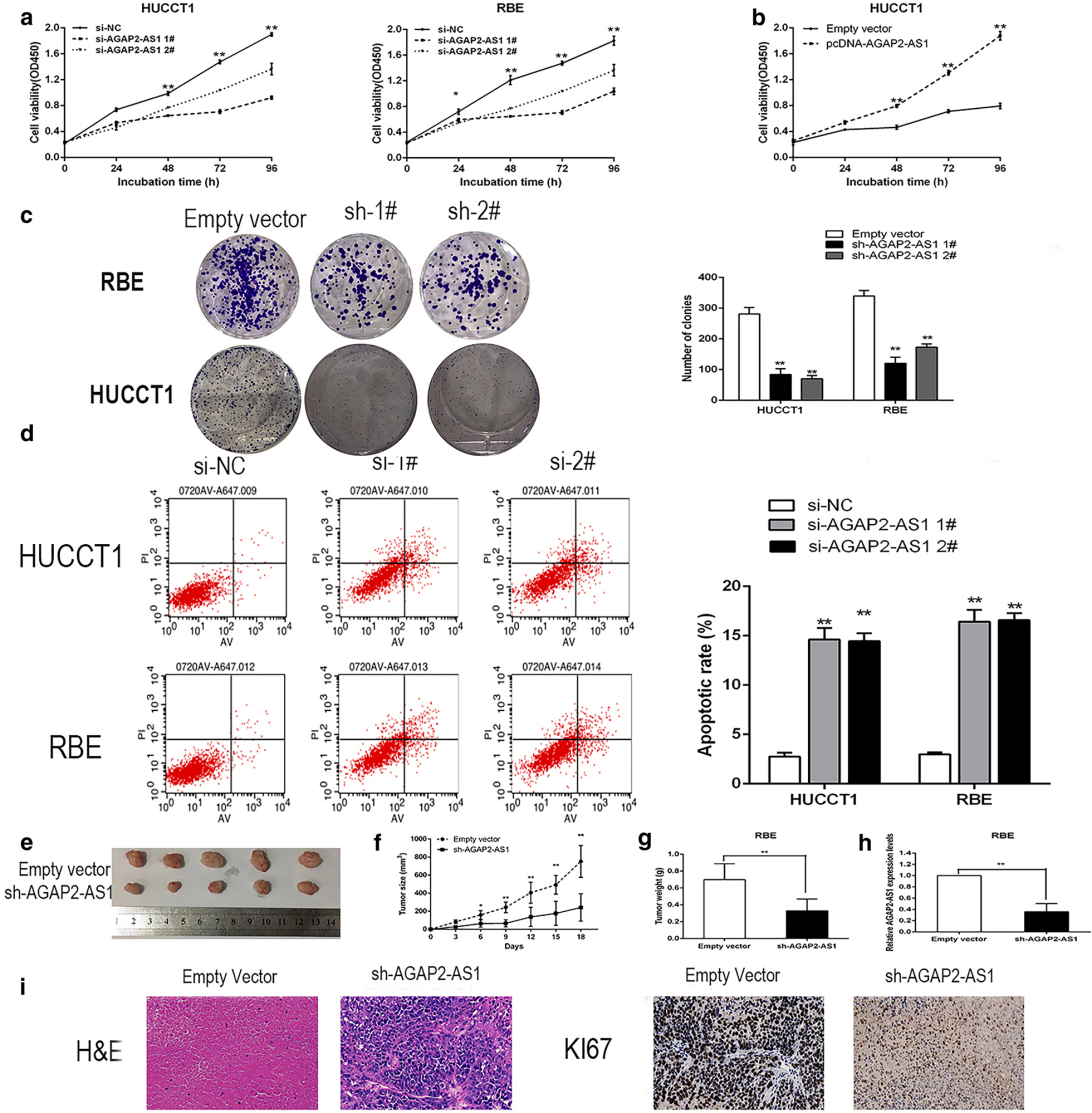
AGAP2-AS1 promotes CCA cell proliferation in vitro and vivo. **a**, **b** CCK-8 assay was performed to determine the proliferation of HUCCT1 and RBE cells with si-AGAP2-AS1 1, 2, pcDNA-AGAP2-AS1, or the negative control. **c** Colony formation assays were performed to determine the proliferation of sh-AGAP2-AS1-transfected HUCCT1 and RBE cells. **d** The percentage of apoptotic cells was determined by flow cytometric analysis. **e** Stable AGAP2-AS1 knockdown were used for the in vivo study. Nude mice carrying tumors from respective groups were shown. **f** Tumor volumes were calculated every 3 days after injection. **g** Tumor weights from two groups are represented. **h** qPCR was performed to detect the average expression of AGAP2-AS1 in xenograft tumors. **i** Images of HE staining and immunohistochemistry in xenograft tumors. Representative Ki-67 protein levels in xenograft tumors as evaluated by IHC. Representative images and data based on three independent experiments. Bars: ± sd, *P < 0.05, **P < 0.01

To assess the biological role of AGAP2-AS1 in CCA, we first investigated the effect of AGAP2-AS1 knockdown on cell proliferation. The CCK8 assay showed that growth of RBE and HUCCT1 cells transfected with si-AGAP2-AS1 was significantly inhibited (Fig. 2a). In contrast, AGAP2-AS1 overexpression promoted cell growth ability (Fig. 2b). Similarly, the results of the colony formation assay showed that AGAP2-AS1 knockdown reduced colony formation and survival in HUCCT1 and RBE cells (Fig. 2c). Flow cytometry assay were used to detect the effect of AGAP2-AS1 knockdown on the proliferation of CCA cells by affecting apoptosis. The proportion of apoptotic cells with AGAP2-AS1 siRNA was significantly increased compared to apoptotic cells induced by scrambled controls (Fig. 2d). Taken together, these data indicate that AGAP2-AS1 drives CCA cell proliferation by inhibiting apoptosis.

### Downregulation of AGAP2-AS1 inhibits CCA tumorigenesis in vivo

To further investigate the effect of AGAP2-AS1 expression levels on tumors in vivo, RBE cells stably transfected with sh-AGAP2-AS1 and empty vector were inoculated into male nude mice. On the 18th day after injection, all mice showed xenotransplantation at the injection site, and the tumor size in sh-AGAP2-AS1 group was significantly lower than that in control group (Fig. 2e). In addition, tumor growth in the sh-AGAP2-AS1 group was significantly slower than that in the control group (Fig. 2f). Furthermore, the mean weight of sh-AGAP2-AS1 tumors was significantly reduced compared to the control group (Fig. 2g). We then used qRT-PCR analysis to show that the mean expression of AGAP2-AS1 in the tumor tissue of the sh-AGAP2-AS1 group was lower than that of the control group (Fig. 2h). Immunohistochemistry confirmed that tumors formed by RBE / sh-AGAP2-AS1 cells showed lower Ki-67 staining intensity than tumors formed by empty vector transfected cells (Fig. 2i). These results further confirmed that AGAP2-AS1 participates in the development of CCA through its effect on the proliferation of CCA cells; inhibition of AGAP2-AS1 expression leads to a decrease in the growth of CCA cells.

### AGAP2-AS1 influences CDKN1A transcription by interacting with EZH2 in CCA cells

**Fig. 3:**
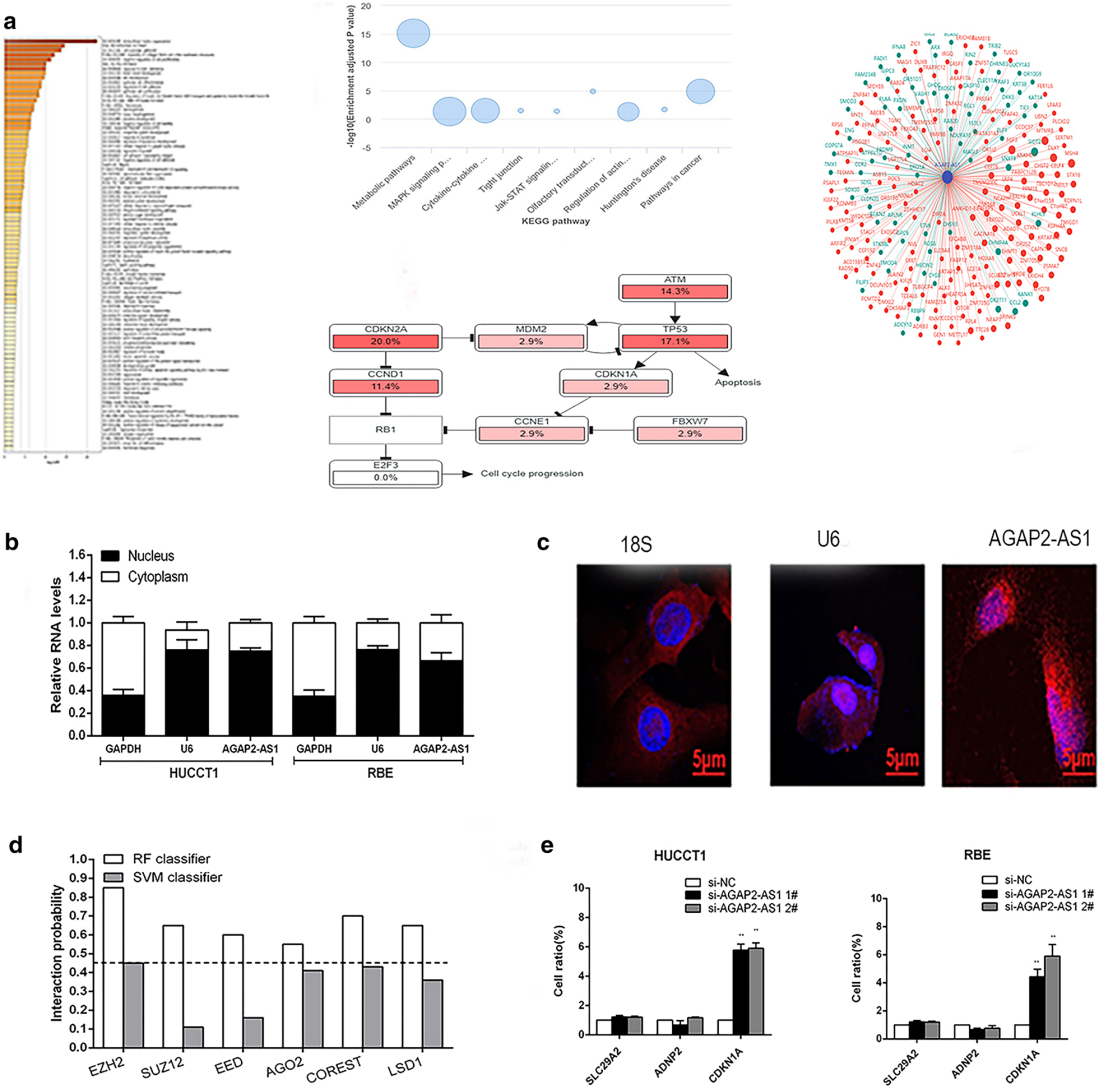
AGAP2-AS1 pathway analysis. **a** GO/KEGG Pathway analysis. **b**, **c** AGAP2-AS1 expression in cell nucleus or cytoplasm of HUCCT1 and RBE cells was investigated by qRT-PCR. U6 was used as a nuclear marker, and GAPDH was used as a cytosol marker. **d** Bioinformatics prediction of RBP. **e** CDKN1A expression was determined by qRT-PCR. Bars: ± sd, *P < 0.05, **P < 0.01

**Fig. 4:**
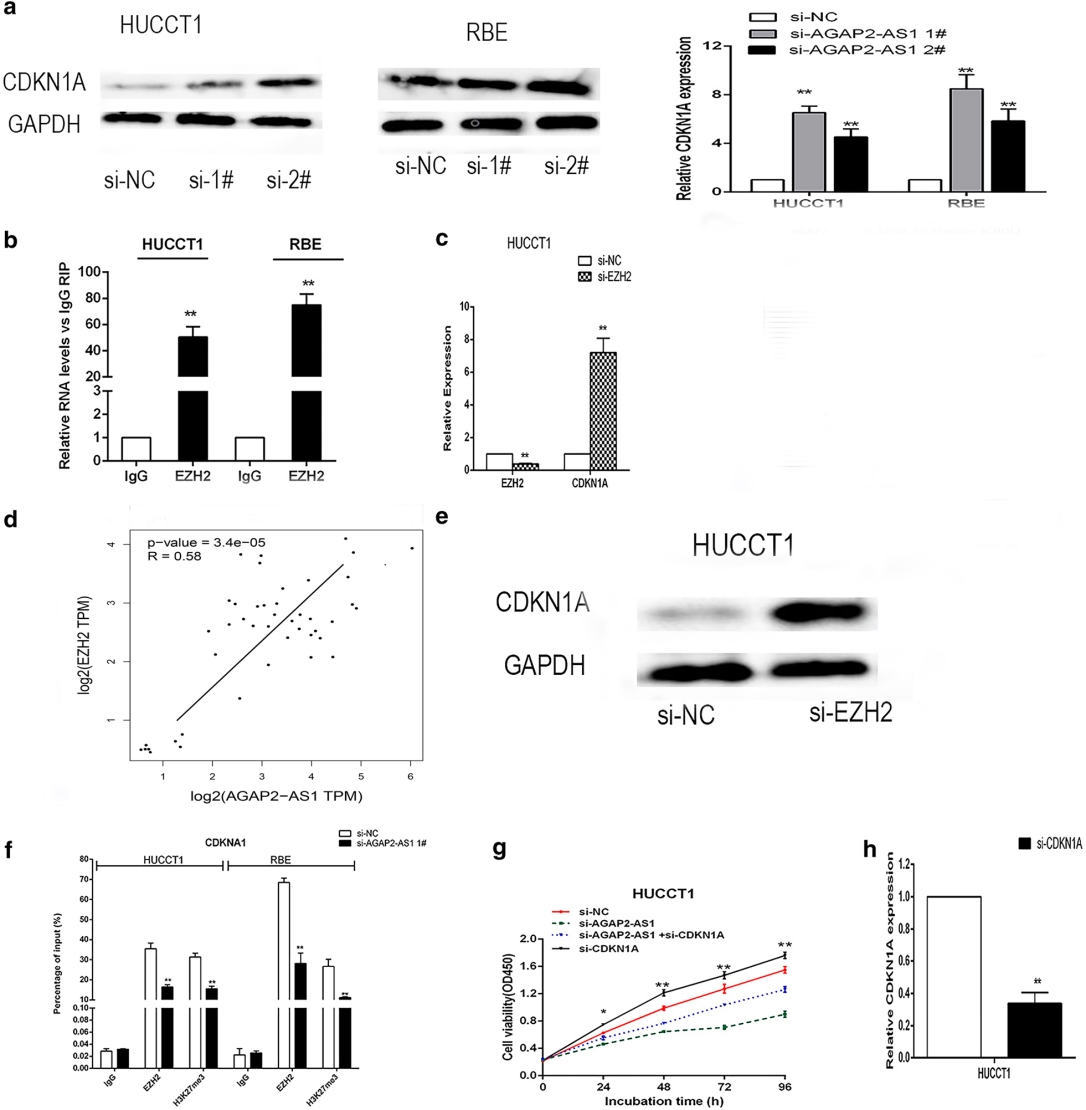
AGAP2-AS1 epigenetically silences CDKN1A transcription by binding to EZH2. **a** CDKN1A expression was determined by qRT-PCR and Western blot assays after AGAP2-AS1 knockdown by transfection. **b** RIP assays were performed in HUCCT1and RBE cells and the coprecipitated RNA was subjected to qRT-PCR for AGAP2-AS1. **c**, **d** Bioinformatics predicts that there is a positive correlation between AGAP2-AS1 and EZH2 and a negative correlation between EZH2 and CDKN1A. **e** Western Blot assays were performed to determine the expression of EZH2 in HUCCT1 cells after EZH2 knockdown. **f** ChIP-qRT-PCR for EZH2 occupancy and H3K27me3 binding to the CDKN1A promoter in HUCCT1 and RBE cells. **g**, **h** CCK-8 assays were used to determine the viability of si-AGAP2-AS1 and si-CDKN1A co-transfected HUCCT1 cells. Representative images and data based on three independent experiments. Bars: ± sd, *P < 0.05, **P < 0.01

To explore the molecular mechanism of the role of AGAP2-AS1 in CCA cell phenotype, by analyzing the KEGG/GO pathway, enrichment gene analysis, to screen genes associated with AGAP2-AS1(https://metascape.org) (Fig. 3a). LncRNA can play a regulatory role by binding RBPs, so we predict some common RBPs. Bioinformatics predicts AGAP2-AS1 binding to EZH2(Fig. 3b).The subcellular localization of cells using fractionation assays and RNA fluorescence in situ hybridization in CCA cells. The results showed that the expression of AGAP2-AS1 in the nucleus was higher than that in the cytoplasm, indicating that it can act as a regulator of transcriptional levels (Fig. 3c, d). To further investigate the potential targets involved in CCA cell proliferation, we predicted some genes by By cBioPortal (https://www.cbioportal.org/). related to proliferation and apoptosis of cancer cells and detected the changes of downstream target expression in CCA cells after further knockdown of AGAP2-AS1,including CDKN1A,ADNP2,SLC29A2 by qRT-PCR (Fig. 3e). Western blot results showed that down-regulation of AGAP2-AS1 significantly increased CDKN1A expression compared to control cells (Fig. 4a). Previous studies have shown that lncRNAs and PRC2 recruit promoters of target genes and their effects on downstream target expression. We predicted EZH2 for RNA immunoprecipitation experiments. We confirmed that AGAP2-AS1 directly binds to EZH2 in HUCCT1 and RBE cells (Fig. 4b).And bioinformatics predicts that there is a positive correlation between AGAP2-AS1 and EZH2 and a negative correlation between EZH2 and CDKN1A (Fig. 4c–e). To further determine whether AGAP2-AS1 silences CDKN1A transcription by recruiting EZH2 to the CDKN1A promoter region, we designed CDKN1A primers in the promoter region and performed chromatin immunoprecipitation assays. The results indicate that EZH2 binds to the CDKN1A promoter region, and knockdown of AGAP2-AS1 reduces binding of EZH2 to the CDKN1A promoter region (Fig. 4f). We also performed rescue assays to determine whether CDKN1A is involved in AGAP2-AS1-mediated CCA cell proliferation. HUCCT1 cells were co-transfected with AGAP2-AS1 and CDKN1A siRNA. CCK8 assays showed that proliferation of HUCCT1 cells co-transfected with si-AGAP2-AS1 and si-CDKN1A was increased compared to that in HUCCT1 cells treated with si-AGAP2-AS1 alone (Fig. 4g, h). These data indicate that AGAP2-AS1 promotes CCA cell proliferation through an epigenetic silencing portion of EZH2-binding CDKN1A transcription. However, further research is needed to identify other possible goals and mechanisms.

## Discussion

Many lncRNAs have been confirmed to be abnormally expressed in tumors such as colorectal cancer, gastric cancer, liver cancer, and prostate cancer (Zhao et al. 2018a; Yoon et al. 2012; Zhou et al. 2018). LncRNAs has the characteristics of poor conservation among species and high specificity of cell expression, which complicates the study of its structure and molecular mechanism (Zou et al. 2018; Lin et al. 2016). For example, The research of our research group shows that lncRNA SPRY4-IT1 may promote estrogen receptor (−) human breast cancer cells by upregulating the expression of zinc-containing proteins (Shi et al. 2015). LncRNA CRNDE promotes colorectal cancer cell proliferation by inhibiting the expression of DUSP5 and CDKN1A (Ding et al. 2017).

Since lncRNA is also an effector molecule, its expression level may be more reflective of the essential characteristics of the tumor; thus, lncRNA as a marker may be superior to mRNA at the tumor diagnosis level (Li et al. 2019). Although many of its abnormalities are known to be expressed in human cancer, the related molecular mechanisms are not fully understood. It is reported that lncRNA can affect the biological function of cancer cells by regulating the expression of target genes (Barbagallo et al. 2018). In this experiment, In addition, we found that the transcription factor SP1 can induce AGAP2-AS1,then we studied the expression of related target genes after knockdown of AGAP2-AS1 in CCA cells, and found that the expression of tumor suppressor gene CDKN1A was significantly increased after knockdown of AGAP2-AS1. LncRNA can regulate gene expression in a variety of ways. For example, binding EZH2 regulates target genes (Munteanu et al. 2018). To confirm the regulatory mechanism of AGAP2-AS1, we performed RNA immunoprecipitation experiments and found that can regulate CDKN1A expression through AGAP2-AS1 and EZH2-mediated histone modifications. CDKN1A, acts as a tumor suppressor in many cancers, where we found that CDKN1A is a downstream regulator of AGAP2-AS1-mediated CCA cell growth arrest. Rescue experiments showed that CDKN1A inhibition may contribute to the carcinogenic function of AGAP2-AS1.

Our study provides a new perspective for AGAP2-AS1 can promote the proliferation of CCA cells,enriches the regulatory network of CCA, and further discovers that identifying new CCA-related lncRNAs has a molecular mechanism for improving the development of CCA and providing clinical therapeutic targets. However, other possible mechanisms by which AGAP2-AS1 participates in CCA remain to be fully understood, and this conclusion still requires more tissue specimen validation.

## Conclusions

We found that AGAP2-AS1 is a LncRNA that up-regulated in CCA tissues, and high expression of AGAP2-AS1 is closely related to the poor prognosis of CCA. It was found in vitro that AGAP2-AS1 promotes proliferation of CCA cells by silencing CDKN1A expression.

## Supplementary information

**Additional file 1: Table S1.** The list of primers and the sequence of siRNAs.

## Abbreviations

LncRNAs: Long non-coding RNA
CCA: Cholangiocarcinoma
RBP: RNA binding protein
TBST: Tris-buffered saline with Tween
