# Supplementary material for: SP1 induced long non-coding RNA AGAP2-AS1 promotes cholangiocarcinoma proliferation via silencing of CDKN1A
Source: Mol Med. 2021 Feb 1;27:10. doi: 10.1186/s10020-020-00222-x (PMC7852216; doi:10.1186/s10020-020-00222-x)
Supplement: Supplementary file 1 — Additional file 1: Table S1. The list of primers and the sequence of siRNAs. [file 10020_2020_222_MOESM1_ESM.docx]

| **Table S1:The list of primers and the sequence of siRNAs** | | |
| --- | --- | --- |
| GENE | Forward primer | Reverse primer |
| AGAP2-AS1 | TACCTTGACCTTGCTGCTCTC | TGTCCCTTAATGACCCCATCC |
| EZH2 | TGCACATCCTGACTTCTGTG | AAGGGCATTCACCAACTCC |
| CDKN1A | AAGTCAGTTCCTTGTGGAGCC | GGTTCTGACGGACATCCCCA |
| GAPDH | AGCCACATCGCTCAGACAC | GCCCAATACGACCAAATCC |
| Sp1 | TGGCAGCAGTACCAATGGC | TAAAGCTTAACCGGTCCCA |
| U6 | ATTGGAACGATACAGAGAAGATT | GGAACGCTTCACGAATTTG |
| ChIP-qPCR primers |  |  |
| CDKN1A | GGTGTCTAGGTGCTCCAGGT | GCACTCTCCAGGAGGACACA |
| AGAP2-AS1 | AACCCCAGTACCCCATCTTG | GGTAATTGGGGAGGGGAGAG |
| Sequences for siRNAs |  |  |
| si-AGAP2-AS1 1# | CCACTCCACCTCAAACTCTTACCTT |  |
| si-AGAP2-AS1 2# | GGGTCATTAAGGGACAGAGTTCAAG |  |
| si-EZH2 | GAGGUUCAGACGAGCUGAUUU |  |
| Si-SP1 | GCCAATAGC  TACTCAACTA  GCCAATAGC  TACTCAACTA  GCCAATAGCTACTCAACTA |  |
| Sh-AGAP2-AS1 | CACCGCCACTCCACCTCAAACTCTTACCTTCGAAAAGGTAAGAGTTTGAGGTGGA CACCGCCACTCCACCTCAAACTCTTACCTTCGAAAAGGTAAGAGTTTGAGGTGGAGTGG GTGG  CACCGCCACTCCACCTCAAACTCTTACCTTCGAAAAGGTAAGAGTTTGAGGTGGAGTGG |  |
| Sh-AGAP2-AS1 | CACCGGGTCATTAAGGGACAGAGTTCAAGCGAACTTGAACTCTGTCCCTTAATGACCC |  |
| Sh-NC | CAGGTGGACTCACAATTCCAAATAT |  |
